# Burden of premature mortality in rural Vietnam from 1999 – 2003: analyses from a Demographic Surveillance Site

**DOI:** 10.1186/1478-7954-4-9

**Published:** 2006-08-08

**Authors:** Dao Lan Huong, Hoang Van Minh, Theo Vos, Urban Janlert, Do Duc Van, Peter Byass

**Affiliations:** 1 Health Strategy and Policy Institute, Ministry of Health, Vietnam; 2 Public Health Faculty, Hanoi Medical University, Vietnam; 3 School of Population Health, Queensland University, Australia; 4 Umeå International School of Public Health, Epidemiology, Department of Public Health and Clinical Medicine, Umeå University, Sweden; 5 Viet Duc Surgery Hospital, Hanoi Medical University, Vietnam

## Abstract

**Background:**

Assessing the burden of disease contributes towards evidence-based allocation of limited health resources. However, such measures are not yet commonly available in Vietnam. Taking advantage of the FilaBavi Demographic Surveillance Site (FilaBavi DSS) in Vietnam, this study aimed to establish the feasibility of applying the Years of Life Lost (YLL) technique in the context of a defined DSS, and to estimate the importance of the principal causes of premature mortality in a rural area of Vietnam between 1999 and 2003.

**Methods:**

Global Burden of Disease methods were applied. Causes of death were ascertained by verbal autopsy.

**Results:**

In five years, 1,240 deaths occurred and for 1,220 cases cause of death information from verbal autopsy was available. Life expectancy at birth was 71.0 (95% confidence interval 69.9–72.1) in males and 80.9 (79.9–81.9) in females. The discounted, but not age weighted YLL per 1,000 population was 85 and 55 for males and females, respectively. The leading causes of YLL and death counts were cardiovascular diseases, malignant neoplasms, unintentional injuries, and neonatal causes. Males contributed 54% of total deaths and 59% of YLL. Males experienced higher YLL than women across all causes. Filabavi mortality estimates are considerably lower than 2002 WHO country estimates for Vietnam. Also the FilaBavi cause distribution varies considerably from the WHO result.

**Conclusion:**

The combination of localised demographic surveillance, verbal autopsy and the application of YLL methods enable new insights into the magnitude and importance of significant public health issues in settings where evidence for planning is otherwise scarce. Local mortality data vary considerably from the WHO model-based estimates.

## Background

Numbers of death and mortality rates are among the simplest indicators for presenting mortality. However, crude or adjusted mortality rates are highly influenced by the health problems of the more advanced age groups at which most deaths occur [1]. While simple death rates constitute an important measure of public health, deaths at younger ages may be considered of greater public health concern than deaths at older ages. Years of Life Lost (YLL), the mortality component of the DALY (Disability Adjusted Life Years), give greater value to deaths of young people, thus emphasising the concept of premature death [2]. The DALY is a health gap measure that combines both time lost due to premature mortality (YLL) and non-fatal conditions (Years Lived with Disability – YLD). This measure was used in Global Burden of Disease and Injury study (GBD), a joint study between the World Bank, the World Health Organization (WHO) and Harvard School of Public Health, with the objective to quantify the burden of disease and injury on human populations and redefine the world's main health challenges [2]. YLL can be disaggregated by cause, age and sex making it relevant for policy makers. In DALY calculations, the YLL component is less contentious than YLD since it simply concerns premature death in relation to life expectancy from a standard life table [3].

In Vietnam, the collection of vital statistical data is weak. The Annual Health Statistical Year Book published by the Ministry of Health presents the leading causes of death, but data are mostly gathered from hospitals and do not represent mortality in the wider community. The current information about mortality by cause, thus, has limited use for health policymaking and priority setting. A recent WHO publication on the global status of causes of death, which reviewed data from 115 country members, had no information from Vietnam [4]. Some initiatives have identified mortality patterns in Vietnam, but only for selected causes such as maternal mortality [5].

A burden of disease assessment requires detailed information about age and cause of death on an individual basis, which often is difficult to obtain in low-income countries. Most estimates for YLL and DALY for these countries have been drawn from models rather than original data. Taking advantage of the ongoing Demographic Surveillance Site (DSS) established in Bavi District, Vietnam, known as FilaBavi [6], this study aimed to estimate YLL by cause in a rural area of Vietnam between 1999 and 2003.

## Methodology

The FilaBavi DSS is located in Bavi district, Hatay province, 60 km to the northwest of the capital, Hanoi. The district is typical of the Red River Delta Region in northern Vietnam. It is a predominantly rural area, in which most people rely on agricultural production. The district population is approximately 250,000, mainly from the Kinh ethnic group (91%) with some other minorities including Muong (8%), Dao, Tay, Hoa, and Khme. Health services are provided through Bavi district health centre in Bavi town, by commune health stations in all its 32 communes and via some private practitioners.

An original sample was selected randomly with probability proportional to population size in the communities, covering the range of geographical regions in the district (including lowland, highland, and mountainous area). Clusters of hamlets or village sub-divisions were the sampling unit. The sample included 67 out of 352 clusters with a total population of about 51,000 inhabitants, and an estimated 11,300 households. The introduction to the site, its routine data collection and management have been described elsewhere [6].

Life expectancy at birth (with 95% confidence intervals) for males and females was calculated based on abridged life tables constructed using the revised Chiang methodology as advised by the Office for National Statistics, United Kingdom [7].

Verbal autopsy (VA) has been used routinely to derive the cause of every death occurring in the setting since surveillance started in 1999. The VA procedure has been described in detail elsewhere [8]. Since the year 2000, six field supervisors with medical background such as nurse or assistant doctor have been VA interviewers while previously 39 lay field surveyors were used. Two physicians derived causes of death based on the International Classification of Disease, version 10 [9], using transcripts of the verbal autopsy interview. A single underlying cause of death was assigned to each case.

The VA diagnoses were then entered into the FilaBavi database and linked to socio-economic information for the deceased and information about the whole population under consideration as denominators for analysis. Data were analysed using Stata 8.0 software.

For measuring the burden of premature mortality, the Standard Expected Years of Life Lost (SEYLL) method was applied from the Global Burden of Disease Study [2] in order to improve the comparability of results between different studies. Discounting but no age weighting was used.

## Results

**Figure 1:**
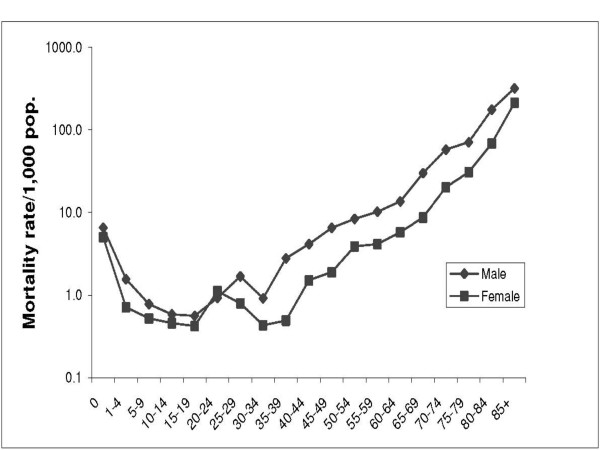
Mortality rate/1,000 population by age group and sex, FilaBavi 1999 – 2003.

Between 1999 and 2003, 1,240 deaths were recorded in FilaBavi from a population base studied of 244,356 person-years, giving a crude mortality rate of 5.1 per 1,000 person-years. Sex-specific rates were 5.7 per 1,000 person-years for males and 4.5 for females. Direct standardisation using the WHO standard population [9] gave a rate of 5.7 per 1,000 person-years, 8.4 for males and 3.9 for females. Life expectancy at birth was 75.7 years overall (95% CI 75.1 – 76.4); 71.0 for males (95% CI 69.9 – 72.1) and 80.8 for females (95% CI 79.9 – 81.9). Males had higher mortality rates than females in all age groups except 20–24 years (Figure 1).

Verbal autopsy information was obtained for 1,220 deaths. Information on the remaining 20 cases is lacking because of migration or failure to find appropriate respondents. Diseases of the circulatory system, malignant neoplasms, infectious diseases and injuries (both unintentional and intentional) were leading causes of death in both males and females (Table 1). Table 2 presents the YLL distribution of the same causes of death by sex showing some differences in the ranking order of the leading causes of YLL, with injuries and infectious diseases swapping ranks.

**Table 1:** Distribution of specific causes of death by sex, FilaBavi 1999 – 2003

**Cause of death**	**Male**		**Female**		**Total**	
	**Nr**	**%**	**Nr**	**%**	**Nr**	**%**
**1. Diseases of the circulatory system**	**193**	**29.4**	**160**	**28.4**	**353**	**28.9**
Stroke	114	17.4	104	18.5	218	17.9
Ischaemic heart disease	12	1.8	13	2.3	25	2.1
**2. Neoplasms**	**101**	**15.4**	**76**	**13.5**	**177**	**14.5**
Malignant neoplasms of digestive organs	55	8.4	40	7.1	95	7.8
Malignant neoplasms of respiratory and intrathoracic organs	24	3.7	8	1.4	32	2.6
**3. Infectious and parasitic diseases**	**80**	**12.2**	**57**	**10.1**	**137**	**11.2**
Pneumonia and lower respiratory infections	38	5.8	21	3.7	59	4.8
Tuberculosis	17	2.6	6	1.1	23	1.9
**4. Injuries**	**75**	**11.4**	**44**	**7.8**	**119**	**9.8**
Drowning	30	4.6	15	2.7	45	3.7
Transport accident	16	2.4	5	0.9	21	1.7
Other unintentional injuries	23	3.5	21	3.7	44	3.6
Intentional injuries	6	0.9	3	0.5	9	0.7
**5. Diseases of Digestive system**	**48**	**7.3**	**23**	**4.1**	**71**	**5.8**
**6. Perinatal conditions**	**37**	**5.6**	**22**	**3.9**	**59**	**4.8**
Low birth weight	17	2.6	9	1.6	26	2.1
Congenital malformation	11	1.7	5	0.9	16	1.3
**7. Diseases of Respiratory system**	**20**	**3.0**	**17**	**3.0**	**37**	**3.0**
**8. Diseases of Genito-urinary system**	**21**	**3.2**	**10**	**1.8**	**31**	**2.5**
**9. Diseases of nervous system**	**10**	**1.5**	**16**	**2.8**	**26**	**2.1**
**10. Complication of labour and delivery**	**-**		**2**	**0.4**	**2**	**0.2**
**11. Others**	**5**	**0.8**	**19**	**3.4**	**24**	**2.0**
**12. Unknown**	**67**	**10.2**	**117**	**20.8**	**184**	**15.1**
**Total**	**657**	**100**	**563**	**100**	**1,220**	**100**

**Table 2:** Years of Life Lost by specific causes of death and sex, FilaBavi 1999 – 2003

**Cause of death**	**Male**		**Female**		**Total**	
	**Nr**	**%**	**Nr**	**%**	**Nr**	**%**
**1. Diseases of the circulatory system**	**2170**	**22.8**	**1587**	**23.7**	**3757**	**23.2**
Stroke	1199	12.6	965	14.4	2164	13.4
Ischaemic heart disease	174	1.8	148	2.2	323	2.0
**2. Neoplasms**	**1548**	**16.3**	**1184**	**17.7**	**2732**	**16.9**
Malignant neoplasms of digestive organs	832	8.7	584	8.7	1416	8.7
Malignant neoplasms of respiratory and intrathoracic organs	319	3.4	118	1.8	437	2.7
**3. Infectious and parasitic diseases**	**1177**	**12.4**	**774**	**11.6**	**1951**	**12.0**
Pneumonia and lower respiratory infections	493	5.2	224	3.3	717	4.4
Tuberculosis	222	2.3	61	0.9	283	1.7
**4. Injuries**	**1703**	**17.9**	**785**	**11.7**	**2488**	**15.4**
Drowning	821	8.6	419	6.3	1240	7.7
Transport accident	349	3.7	120	1.8	469	2.9
Other unintentional injuries	407	4.3	178	2.7	585	3.6
Intentional injuries	126	1.3	76	1.1	202	1.2
**5. Diseases of Digestive system**	**727**	**7.6**	**293**	**4.4**	**1020**	**6.3**
**6. Conditions originating in the perinatal period**	**1121**	**11.8**	**671**	**10.0**	**1792**	**11.1**
Low birth weight	515	5.4	275	4.1	790	4.9
Congenital malformation	333	3.5	152	2.3	485	3.0
**7. Diseases of Respiratory system**	**217**	**2.3**	**129**	**1.9**	**346**	**2.1**
**8. Diseases of Genito-urinary system**	**348**	**3.7**	**166**	**2.5**	**514**	**3.2**
**9. Diseases of nervous system**	**182**	**1.9**	**206**	**3.1**	**388**	**2.4**
**10. Complication of labour and delivery**		**-**	**53**	**0.8**	**53**	**0.3**
**11. Others**	**53**	**0.6**	**260**	**3.9**	**313**	**1.9**
**12. Unknown**	**618**	**6.5**	**803**	**12.0**	**1421**	**8.8**
**Total**	**9516**	**100**	**6692**	**100**	**16208**	**100**

**Figure 2:**
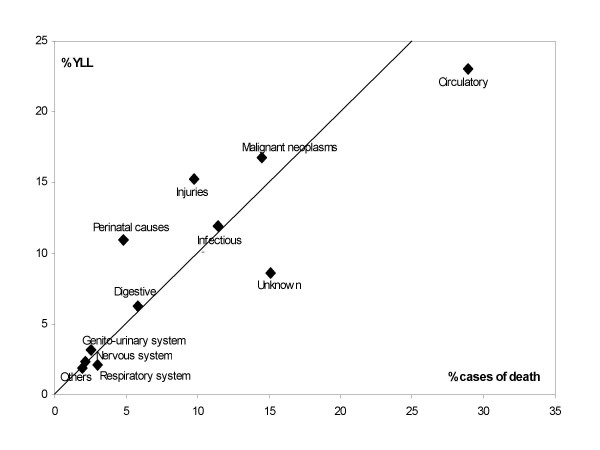
Years of life lost (YLL) versus crude mortality, FilaBavi 1999 – 2003.

The proportions of YLL and the corresponding cause-specific proportions of mortality for the same 1,220 deaths are presented in Figure 2. There are substantial differences evident between these two approaches of describing cause-specific mortality. Points to the left of the line of equality (y = x) indicate causes that contribute to YLL more than to deaths. Many points lie close to the line of equality. Infectious diseases, diseases of genito-urinary system, respiratory system, and nervous system are represented by similar percentages of YLL and mortality. External causes and perinatal conditions are more prominent contributors to YLL as they occur at younger ages while cardiovascular diseases and the ill-defined category of deaths largely affect the elderly.

**Figure 3:**
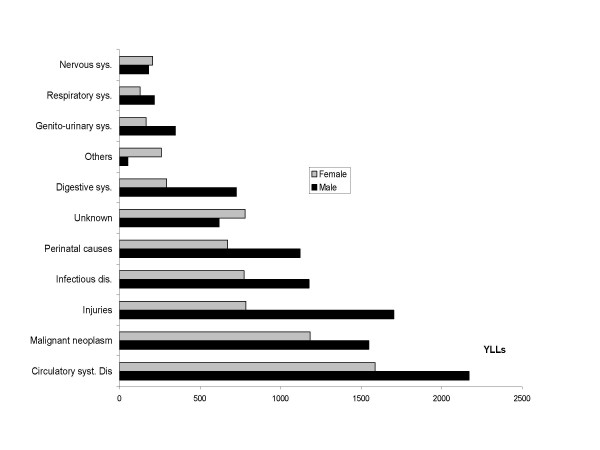
Total YLL by cause of death for males and females, FilaBavi 1999 – 2003.

Men also experienced higher YLL than women for all causes except miscellaneous and unknown causes (Figure 3). Cardiovascular diseases and unintentional injuries contributed most to YLL for men while malignant neoplasms and cardiovascular diseases ranked first and second for women.

Table 3 compares the age-standardised rates per 100,000 population by specific cause of death estimated by the WHO for Vietnam in 2002 [10], and the FilaBavi data. Remarkable differences were observed; Filabavi recorded much less deaths from ischaemic heart disease, infectious diseases and chronic respiratory disease but a much higher death rate from drowning.

**Table 3:** Age standardised mortality rate per 100,000 populations by specific cause of death: the WHO estimation for Vietnam in 2002 [10] and FilaBavi data 1999 – 2003

**Cause of death**	**Rate per 100,000 populations**	
	
	**WHO estimate – 2002**	**FilaBavi 1999 – 2003**
**1. Disease of the circulatory system**	**199**	**147**
Stroke	73	91
Ischaemic heart disease	82	10
**2. Neoplasms**	**80**	**74**
Malignant neoplasms of digestive organs	34	39
Malignant neoplasms of respiratory and intrathoracic organ	13	13
**3. Infectious and parasitic disease**	**122**	**58**
Pneumonia and lower respiratory infections	33	25
Tuberculosis	22	10
**4. External causes**	**60**	**50**
Drowning	5	19
Transport accident	15	9
Other unintentional injuries	24	18
Intentional injuries	15	4
**5. Diseases of Digestive system**	**24**	**30**
**6. Perinatal conditions**	30	25
Low birth weight	14	11
Congenital malformation	6	7
**7. Diseases of respiratory system**	**64**	**15**
**8. Disease of genitor urinary system**	**13**	**13**
**9. Disease of nervous system**	**17**	**11**
**10. Complication of labour and delivery**	**3**	**1**
**11. Others**	**25**	**10**
**Overall**	**640**	**570**

## Discussion

### The burden of premature mortality in rural Vietnam

The life expectancy at birth for the Filabavi population during the five-year period was similar to a previous estimate using three-year data from 1999 to 2001 [11]. This result was considerably higher than the 2002 WHO estimate for Vietnam of 67.1 (95% CI 66.3 – 68.1) in men and 72.2 (95% CI 71.4 – 73.1) in women [10]. For all causes of death combined, the rate of YLL in FilaBavi was 85/1,000 population and 55/1,000 population for males and females, respectively. These figures were lower than the WHO estimation for premature mortality in Vietnam in 2002, i.e. 110.7 YLL/1,000 population for males and 84.7 YLL/1,000 population for females [10]. We are confident of capturing all deaths in the DSS. A validation study carried out in FilaBavi showed that the formal commune population registration system under-registered 19% of deaths, and the re-census (equivalent to a cross sectional survey) missed 4% deaths in the area, while the FilaBavi DSS only missed one death in the same period [12]. It is assumed that the North Delta Region, of which FilaBavi is a part experiences lower mortality than the rest of the country. However, it is unlikely that the difference in mortality between what we have recorded in FilaBavi and the true mortality experience in the whole of Vietnam is as large as the gap between the Filabavi and WHO estimates would indicate.

In the Global Burden of Disease Study [2], YLL was estimated for all WHO Member states. The Vietnam estimates were based on modelling approaches rather than original data. The WHO estimation for Vietnam in 2002 indicated that the leading causes of YLL were perinatal conditions followed by ischaemic heart disease (IHD) and stroke, while the leading causes of mortality were IHD, stroke, and chronic obstructive pulmonary disease (COPD) [10]. Our estimates show a rather different pattern. First, the Filabavi recorded infant mortality rate over the five-year period was 19.3/1,000 live births, similar to a UNICEF estimate (18 per 1,000 live births in 2003), and lower than in neighbouring countries such as Thailand, the Philippines, and Indonesia [13], despite their superior economic performance. The WHO estimate is a bit higher than that (21 per 1,000 live births in 2003) [14] and hence gives a more prominent position to perinatal conditions in their ranking of YLL. Second, stroke is a much greater cause of death in Filabavi than IHD while WHO estimates the reverse (table 3). Third, of the other causes and groups of conditions listed in tables 1 and 2, as well as in table 3 for mortality rates, pneumonia, road traffic accidents and cancers are responsible for a similar proportion of overall mortality but the WHO estimates for drowning are much lower and for COPD are much higher.

The low mortality rate from infectious disease in FilaBavi compared to the WHO estimate could reflect a different pattern by geographical region in the country, where mountainous area would have more epidemics like malaria, dengue fever, etc than the delta region like FilaBavi [15].

Data from the Vietnam Multi-Center Injury Survey (VMIS) showed injury deaths accounted for 52% of potential years of life lost before age 65, with 38% lost due to drowning and 28% due to transport-related deaths [16]. Another source of national data also showed that injuries are the biggest killer of children, accounting for 70% of mortality among under 20 year-olds, mainly from drowning [17]. The leading sub-causes in our dataset were drowning for the birth cohort under 20 years and traffic accidents for adults. Out of the total YLL from injuries, drowning alone accounted for nearly 50% while traffic-related accidents accounted for 18.9%. Globally, a third of drowning is estimated to occur in the Western Pacific Region, which includes Vietnam, where children under five years of age have the highest drowning mortality rate worldwide [18]. The WHO model failed to identify the burden of drowning in Vietnam. The actual mortality rate from drowning in FilaBavi was almost five times higher despite the fact that FilaBavi is a rural area. In this rural setting, the main exposures to childhood injuries are not associated with recreation or leisure but with everyday activities associated with a lack of supervision and unsafe environments such as irrigated rice fields.

### The importance of YLL for health policy making and planning

This study indicates YLL is a useful indicator to present to policy makers next to the more traditional death counts and rates. Although calculating and explaining YLL is more complex than simple mortality rates, it adds value in demonstrating the effect on the population for each individual cause of death [19]. Mortality statistics tend to emphasize causes of death among the elderly, where most deaths occur, and thus give less priority to younger age groups [20]. YLL in turn tends to emphasise those causes of death, which often occur in younger age groups because of their larger potential for future losses. Though it is only one component of DALY approach the YLL are likely to be the dominant component of total burden of disease in Vietnam. In Vietnam in general, and for this setting in particular, YLL approach has not been used previously to assist in health planning, probably due to the lack of available relevant data. The YLL index can indicate priorities for interventions against deaths from specific causes, according to likely future patterns of life lost.

The differences between the modelled WHO rates and the Filabavi observed data might be explained by the fact that Filabavi is a selected small part of the country known to have lower mortality than the rest of the country. A more likely explanation is that the modelled WHO data poorly reflect the true mortality experience in Vietnam and hence, emphasises the need for countries to do their own burden of disease assessments.

### Methodological issues

Our first aim, of assessing the feasibility of the YLL approach in the context of a DSS rather than in a society with universal mortality registration, has to be considered in the context of this example. The dataset used included approximately 250,000 person-years and over 1,000 deaths. The duration of the time period (5 years in this case) was a less relevant consideration, provided that there was a reasonable degree of consistency in mortality patterns over the period observed. We would suggest that other DSS datasets, for example from Indepth sites , could be analysed in this way to highlight mortality patterns of public health significance and to make international comparisons between societies without routine registration. Further analyses linking mortality by cause to risk factor data available in DSS settings, could increase understanding of important public health issues.

This study relied on VA to ascertain cause of death. Hospital-based validation studies have been carried out in various settings [21-23] even though they are not considered to provide the optimal comparison. There is debate about the validity of such studies based on arguments such as interviewer information bias, sample selection, and different cut off point for sensitivity and specificity [24]. However, there is no alternative way to validate verbal autopsy studies in countries with poor vital registration data. The greater concern about the validity of VA diagnosis is the determination of cause of death for disease with no distinctive symptoms, such as chronic and degenerative diseases. This could be partly explained the low rate of COPD in FilaBavi. Proper interviewer training, close field procedure supervisions, and comparison between physician diagnoses and a probabilistic model [25], were efforts to improve the quality of our study. We have not been able to carry out a validation study because of the limited number of deaths reported in the local district hospital (less than 15 cases annually).

In conclusion, the combination of localised demographic surveillance, VA, and the application of YLL methods enable new insights into the magnitude and importance of significant public health issues in settings where evidence for planning is otherwise scarce.

### Ethical considerations

The research Ethics Committee at Umeå University has given ethical approval for the FilaBavi household surveillance system, including data collection on vital statistics reference number 01-420. This specific study was also approved by the Scientific and Ethical Committee for Medical Research, Hanoi Medical University.

## Competing interests

The author(s) declare that they have no competing interests.

## Authors' contributions

DLH was responsible for study design, data collection, statistical analysis, and write the manuscript. HVM participated in the design of the study, data collection, statistical analysis and edited the manuscript. TV contributed in statistical analysis, the interpretation of the results, and edited the manuscript. UJ participated in the design of the study and edited the manuscript. DDV participated in the design of the study, data collection and edited the manuscript. PB participated in the design of the study, the interpretation of the result, and edited the manuscript.
